# RBO in the Web of Science: Internationally Recognized Quality

**DOI:** 10.1055/s-0045-1812090

**Published:** 2025-11-18

**Authors:** Geraldo Motta

**Affiliations:** 1 Sociedade Brasileira de Ortopedia e Traumatologia, São Paulo, SP, Brazil

We are pleased to announce that the *Revista Brasileira de Ortopedia* (RBO) has been indexed in the Web of Science database, maintained by Clarivate Analytics. This achievement represents international recognition of the journal's editorial quality and solidifies RBO's position as a leading reference in Orthopedics and Traumatology across Latin America.

Inclusion in the Web of Science is not an endpoint, but part of a continuous editorial strategy guided by rigorous standards: transparent peer review, timeliness, ethical integrity, internationalization, and scientific relevance. These principles have shaped every step of our journey to this milestone and will continue to direct our future efforts.

This accomplishment extends beyond RBO, it is a collective achievement for the entire Brazilian orthopedic community. By attaining this level of recognition, we enhance the visibility of Brazilian research, foster international collaborations, and strengthen the global presence of our scientific production.

With recognition comes greater responsibility. We are committed to maintaining the highest standards of quality, encouraging original and impactful research, streamlining editorial processes, and continuously updating our practices.

We reaffirm our dedication to excellence and invite our authors, reviewers, and readers to continue contributing to RBO. Now recognized by one of the world's most important scientific databases, RBO remains a space for innovation, credibility, and impact in Orthopedics.

